# Translation, Cultural Adaptation and Psychometric Evaluation of the Transformational Leadership Scale in Intensive Care Unit Nurses: Empirical Research Quantitative

**DOI:** 10.1002/nop2.70587

**Published:** 2026-05-25

**Authors:** Katarzyna Lis, Natalia Sak‐Dankosky, Bożena Czarkowska‐Pączek

**Affiliations:** ^1^ Department of Clinical Nursing Medical University of Warsaw Warsaw Poland

**Keywords:** intensive care nursing, management in nursing, psychometric validation, transformational leadership

## Abstract

**Background:**

Transformational leadership, based on inspiration, motivation and trust‐building within teams, is considered one of the most effective management styles in healthcare. In nursing, transformational leadership promotes open communication, interdisciplinary collaboration and the implementation of preventive measures, which directly impact the quality of care and reduce medical errors.

**Aim(s):**

The aim of this study was to apply the Transformational Leadership Scale to the Polish cultural context and to evaluate its psychometric properties among intensive care nurses.

**Methods:**

A cross‐sectional, descriptive study was conducted to perform the cultural adaptation and psychometric validation of the Transformational Leadership Scale. The study included 433 nurses working in adult intensive care units in Poland, recruited via convenience sampling. Data were collected between March and November 2022. The survey consisted of three parts: (1) general information, (2) the Transformational Leadership Scale and (3) socio‐demographic data. The questionnaire comprised 54 items, divided into two main sections concerning: (1) the immediate supervisor (43 items) and (2) the Director of Nursing (9 items). Responses were recorded using a 5‐point Likert scale. The questionnaire was translated using a forward–backward translation procedure with a monolingual test. Content validity was assessed by experts using the item‐level (I‐CVI) and scale‐level (S‐CVI) content validity indices. Confirmatory factor analysis (CFA) was performed to assess model fit. Reliability was assessed using McDonald's *ω*, Cronbach's *α* and composite reliability (CR), while convergent validity was examined using the average variance extracted (AVE).

**Results:**

Items in the I‐CVI ranged from 0.75 to 1, and the mean score for the S‐CVI was 0.944. CFA confirmed that the five‐factor model with 54 items demonstrated a good fit to the data. The analysis confirmed the high reliability of the subscales (McDonald's *ω* = 0.935 to 0.971, Cronbach's *α* = 0.934 to 0.971, CR = 0.924 to 0.980) and convergent validity (AVE = 0.639 to 0.799).

**Conclusion:**

The Transformational Leadership Scale is a valid, reliable and culturally adapted instrument for measuring transformational leadership among Polish intensive care nurses.

**Implications for the Profession:**

The application of the Transformational Leadership Scale may be valuable for designing and implementing appropriate management strategies to support the development of a transformational leadership style in nursing.

**Impact (Addressing):**

What problem did the study address?

There is no psychometrically correct tool for assessing Transformational Leadership in Poland.

What were the main findings?

The Transformational Leadership Scale is a psychometrically valid tool for critical care nursing.

Where and on whom will the research have an impact?

The results provide a psychometrically correct tool for the assessment of transformational leadership in the environment of critical care nurses in Poland and will complement the knowledge in the country and abroad in the area of transformational leadership.

**Reporting Method:**

The manuscript's authors have adhered to the EQUATOR guidelines, using the STROBE reporting guidelines.

**No Patient or Public Contribution:**

Patients or members of the public were not involved in this study.

## 1 Introduction

Constantly increasing demands for quality care, rising costs and nursing staff shortages are global challenges faced by healthcare systems worldwide (Casida and Parker 2011). The COVID‐19 pandemic highlighted the importance of effective leadership in responding to rapidly evolving crisis situations (Hayes and Cocchi 2022).

Transformational leadership (TL) is recognised as one of the effective leadership approaches for supporting organisational and individual development in a rapidly changing healthcare environment (Fischer 2016). In addition, it was pointed out that charisma, inspiration, intellectual thinking and individual attention are four characteristics of leaders that characterise TL and influence the audience (Çelik Durmuş and Kırca 2020). What distinguishes TL from the traditional (and still widely used) leadership style is: building employee‐leader relationships based on trust and communication, a multidisciplinary approach, empowering, engaging and encouraging creative problem solving by employees and personal development (Tian et al. 2020). Moreover, through strong team collaboration and shared decision‐making, TL enhances employee engagement and fosters identification with organisational values and goals (Doody and Doody 2012).

Effective TL is associated with reduced staff turnover (Gillet et al. 2013), which is particularly important for workforce planning in healthcare institutions and is linked to high organisational costs related to employee turnover (Cameron et al. 2004). Moreover, TL contributes to improved quality of nursing care, increases patient safety and improves job satisfaction among employees (Boamah et al. 2018; Lotfi et al. 2018). What is more, TL may have a positive effect on reducing the level of stress and preventing professional burnout (Guo et al. 2022). Healthcare workers are particularly vulnerable to such problems due to the dynamic and stressful nature of their work (Pishgooie et al. 2019). Furthermore, TL leaders promote autonomy by involving employees in the decision‐making process. This, in turn, increases job satisfaction, commitment and the employee's ability to solve problems or offer solutions independently and creatively, leading to a better quality of care and higher patient satisfaction (Ferreira et al. 2020).

Intensive care units (ICUs) have a high demand for TL. Nursing work in ICUs is associated with the unpredictability of care, rapid patient turnover, the treatment of life‐threatening conditions in patients and the need to provide a highly specialised level of critical care (Brewster et al. 2020). All of this may lead to physical strain and psychological stress, which can negatively affect the health and well‐being of nurses working in the ICU. In addition, it may lead to burnout, moral distress, dissatisfaction, poor performance, staff turnover and ultimately poor quality of clinical care (Aiken et al. 2012; Guttormson et al. 2022).

## 2 Background

Poland has one of the lowest numbers of nurses per capita in the European Union (EU), with 5.1 nurses per 1000 inhabitants and the average age of nurses is 53.2 years (State of Health in the EU 2021). Furthermore, the lack of generational replacement in the nursing workforce and ongoing staff shortages make it difficult to ensure high‐quality nursing care (State of Health in the EU 2021; Report of the Supreme Council of Nurses and Midwives 2022). In Poland, it is emphasised more and more commonly that graduates do not take up work in the nursing profession, as well as that the migration of nurses has increased since joining the EU (Szpakowski et al. 2019). Moreover, studies indicate that ICU nurses in Poland are exposed to excessive workload, which causes rationing of care, reduced job satisfaction, burnout and decreased quality of nursing services (Młynarska et al. 2020; Świątek et al. 2017). This poses a significant logistical challenge for leaders in nursing care coordination.

In the current healthcare context, it is necessary to implement changes in the employee management method to a more efficient and supportive one, because TL may prove effective in case of staff shortages. As a first step, emphasis should be placed on developing a reliable management assessment tool among leaders. This will allow diagnosing and implementing strategies to strengthen TL among healthcare leaders, taking into account a country's cultural context. In addition, cross‐cultural and international collaborative research is needed in nursing due to the global migration of nurses, in which the experiences of nurses educated abroad are an important aspect (Moyce et al. 2016). Addressing this knowledge gap may provide valuable insights for both Polish and international nursing leaders.

In this study, the Transformational Leadership Scale (TLS) tool created by Finnish researchers was used (Eneh et al. 2012). The TLS was developed as part of the ‘Attractive and Safety Hospital Project’, which involved researchers from the University of Eastern Finland and Kuopio University Hospital (Kvist et al. 2013).

The goal of this study is to carry out cross‐cultural adaptation of the Transformational Leadership Scale and analyse its validity and reliability among Polish intensive care nurses.

## 3 Methods

### 3.1 Design

This was a two‐phase cross‐sectional descriptive study.

Phase I: translations, expert committee and pilot testing (monolingual test) to ensure content and face validity.

Phase II: testing the translated questionnaire in a sample of nurses working in the ICU to assess the psychometric properties of the instrument.

The study was reported in accordance with the EQUATOR guidelines, using the STROBE statement for observational studies.

### 3.2 Sample

To ensure the reliability and validity of the research results, it was assumed that each scale item should be tested with at least 5 to 10 respondents per item (in the current study, 8 respondents per item) (Boateng et al. 2018). Furthermore, for analyses using DWLS estimates, a sample size of 200–500 is assumed as acceptable (Bandalos 2014; Forero et al. 2009). In CFA studies, the typical minimum number of participants is *N* = 150–200—a sample size of 433 provided adequate power (> 0.80). The study took into account the possible drop rate. The final percentage of incomplete or rejected questionnaires was 5%.

### 3.3 Study Instrument

The authors of the TLS granted permission for translation and use of the instrument (Eneh et al. 2012). In the original study, an exploratory factor analysis of 54 TLS items identified five factors: Leadership Ethics, Nursing process management, Feedback and rewards, Professional development and Nursing directors (Eneh et al. 2012; Kvist et al. 2013). The instrument demonstrated high reliability in a pilot study conducted in 2007, with Cronbach's alpha ranging from 0.884 to 0.944. Subsequent studies reported Cronbach's alpha values of 0.91–0.97 in 2008 and 0.909–0.968 in 2010 (Eneh et al. 2012; Kvist et al. 2013). The Polish version of the TLS consisted of three parts: (1) Introduction, instructions and basic information about the survey, (2) TLS, (3) Sociodemographic data. Part 1 of the survey contained information explaining the purpose of the survey and the concept of transformational leadership, informed the respondents about the anonymity and voluntariness of the survey, provided instructions for completing the tool and informed how the collected sociodemographic data would be processed. The TLS contained a total of 54 items and was divided into two main sections: (1) My nursing team leader (direct supervisor; ward manager/nurse care coordinator, directly managing daily work)—43 items, (2) Director of Nursing (Head Nurse)—9 items. The TLS identified 5 factors: (1) Leadership ethics, (2) Nursing management, (3) Feedback and rewards, (4) Professional development, (5) Director of nursing. Factors 1–4 referred to the nurses' immediate supervisor (ward nurse/nurse care coordinator) and factor 5 related to the Head Nurse/Director of Nursing. The survey included single‐choice answers which were constructed on a 5‐point Likert scale with the following answers possible: 1—Strongly disagree, 2—Rather disagree, 3—It is hard to say, 4—Rather agree, 5—Strongly agree.

### 3.4 Phase I

#### 3.4.1 Translation and Cross‐Cultural Validity

The forward‐blind backward translation process with monolingual test was used (Maneesriwongul and Dixon 2004; Wild et al. 2005). Both English versions of the instrument were analysed by two independent translators and researchers. The first translator translated the TLS from English into Polish. Then, a second independent translator translated the Polish version into English. Finally, the two translators and the research team members jointly analysed both versions for semantic, conceptual, linguistic and contextual differences to construct the final text (Beaton et al. 2000).

#### 3.4.2 Content and Face Validity

The expert committee consisted of eight ICU nurses, all of whom were professionally active in intensive care units, with professional experience in ICU nursing constituting the inclusion criterion for participation in the expert group. Among them, three nurses had additional experience in nursing staff management (ICU nursing team coordinators), one nurse was a scientist specialising in management and four were nurses working in the ICU. The experts rated the relevance of each item using a four‐point scale according to Lynn (1986): (1) not relevant, (2) somewhat relevant, (3) relevant, (4) very relevant. It was assumed that the acceptable level of agreement among the experts should be at least 0.80 and, ideally, higher than 0.90 (Polit and Beck 2006). To avoid an inflated estimate of validity among experts, the optimal number of experts in this study was determined to be above 7 (8 in this study) (DeVon et al. 2007). First, each expert independently rated each item in the TLS using the Content Validity Index (CVI) approach and subsequently, they discussed their opinions together within a consensus development conference to reach agreement. A content validity index was calculated for each item (I‐CVI) as well as for the overall scale (S‐CVI). Items from the I‐CVI had values ranging from 0.75 to 1 and the score for the S‐CVI was 0.944. None of the experts raised any objection to any of the items. They evaluated the Polish version of the TLS to be representative of the target construct it is intended to measure. The tool was evaluated for grammar, syntax, organisation, appropriateness and logical flow. During the expert discussion, consensus was reached. One of the experts suggested clarifying the ‘director of nursing’ and the ‘immediate supervisor’ terms, because of the Polish substitutes for those terms. Based on this suggestion, additional explanatory information regarding this issue was added to the introductory section of the survey.

### 3.5 Phase II

#### 3.5.1 Setting

The data were collected from March 2022 to November 2022. The inclusion criteria for the study were: having an active licence to practice as a nurse in Poland and at least one year of experience working in an adult ICU (Aydogan and Ulupinar 2020). It was assumed that between 5 and 10 respondents per test item would provide an adequate sample size; 8 respondents per item were included in the present study (Boateng et al. 2018). To ensure a sufficiently large sample, the data were collected partly using the Lime Survey (online platform) and partly using a paper version delivered to nine hospital wards. Conducting surveys electronically in this type of research is fully justified, as it helps to reduce costs and reach the widest possible group of respondents (Jones et al. 2008). The study was anonymous and voluntary, completing the questionnaire and returning it to the researchers or returning it at a designated place in the ward (marked box) meant consent to participate in the study. Prior to the study, approvals had been obtained from clinical department heads to conduct the study at the facilities. The researchers delivered and distributed questionnaires to nurses who were willing to participate and informed them about how to return the questionnaires. The online version of the instrument was made available in the wards and on the websites of the Polish Association of Anesthesiology and Intensive Care Nurses.

#### 3.5.2 Data Analysis

Descriptive statistics were used to characterise the sociodemographic characteristics of the study participants. To establish the structure of the TLS questionnaire, a confirmatory factor analysis (CFA) was performed for the 5‐factor questionnaire structure, which was adopted by the authors of the tool (Eneh et al. 2012). Diagonally weighted least squares (DWLS) was used as an estimation method. The following criteria were used as measures of model adjustment: RMSEA ≤ 0.08; CFI ≥ 0.95; SRMR ≤ 0.08 (Hu and Bentler 1999). The reliability of the subscales was calculated using three indices: McDonald's *ω*, Cronbach's *α* and composite reliability (CR). McDonald's *ω* and Cronbach's *α* > 0.7 were used as criteria for satisfactory reliability, while for CR it was assumed that values > 0.6 indicated acceptable reliability (Fornell and Lacker 1981). In addition, average variance extracted (AVE) analyses were conducted to determine convergent validity. Values > 0.5 indicated convergent validity (Fornell and Lacker 1981). The analyses were carried out in JASP 0.16.4 using the lavaan package.

#### 3.5.3 Ethical Considerations

The survey did not cause physical harm, as the respondents were informed. Stress related to the assessment by the supervisor and fear of possible consequences were identified as potential risks. The use of an online survey, distribution in the wards by the researchers, return to a labelled box and limited participation by the supervisor (consent) were intended to minimise the above‐mentioned risk. The study received approval from the Medical University of Warsaw Committee on Research Ethics (AKBE/49/2022) on February 2, 2022.

## 4 Results

### 4.1 Sample

The study comprised 433 people aged 20 to 63, with work experience from 1 to 45 years, including experience in the ICU of 1 to 39 years. Detailed sample characteristics are presented in Table 1.

**TABLE 1:** Sociodemographic characteristics (*n* = 433).

Variable	*n* (%)	*M* (SD)
Sex		
Female	263 (60.7)	—
Male	55 (12.7)	—
Missing data	115 (26.6)	—
Age	—	38.52 (9.91)
Years of working experience	—	15.41 (10.60)
Years of working experience in ICU	—	11.47 (9.17)
Education		
Nursing vocational/high school diploma	24 (5.5)	—
Bachelor's in nursing science	112 (25.9)	—
Master's in nursing science	178 (41.1)	—
Doctoral degree	4 (0.9)	—
Postgraduate education		
Specialist course	225 (52.0)	—
Special qualifications course	186 (43.0)	—
Qualification course (3‐month intensive care nursing qualification course)	119 (64.0)	—
Nursing specialty	194 (44.8)	—
Specialty course (2‐ year intensive care nursing specialty course)	146 (75.2)	—
Inter‐hospital nursing courses	70 (16.2)	—
How many patients do you take care of during your shift?	—	2.98 (2.30)
Type of employment		
Fixed‐term traditional job (fixed‐term or permanent)	259 (59.8)	—
Other types of contract	59 (13.6)	—
Working mode		
Morning shift (7.5 h)	19 (4.4)	—
Day/Night shift (12 h)	239 (55.2)	—
Day‐night shift (24 h)	60 (13.9)	—
Average number of working hours in a month	—	187.67 (51.39)
Kind of hospital		
City/county hospital	101 (23.3)	—
Provincial hospital	68 (15.7)	—
Teaching hospital/University/Research institute	143 (33.0)	—
Other	6 (1.4)	—

Abbreviations: %, percentage; *M*, mean; *n*, number (*n* does not always add up to 433 in all variables due to missing items); SD, standard deviation.

### 4.2 Construct Validity

**FIGURE 1:**
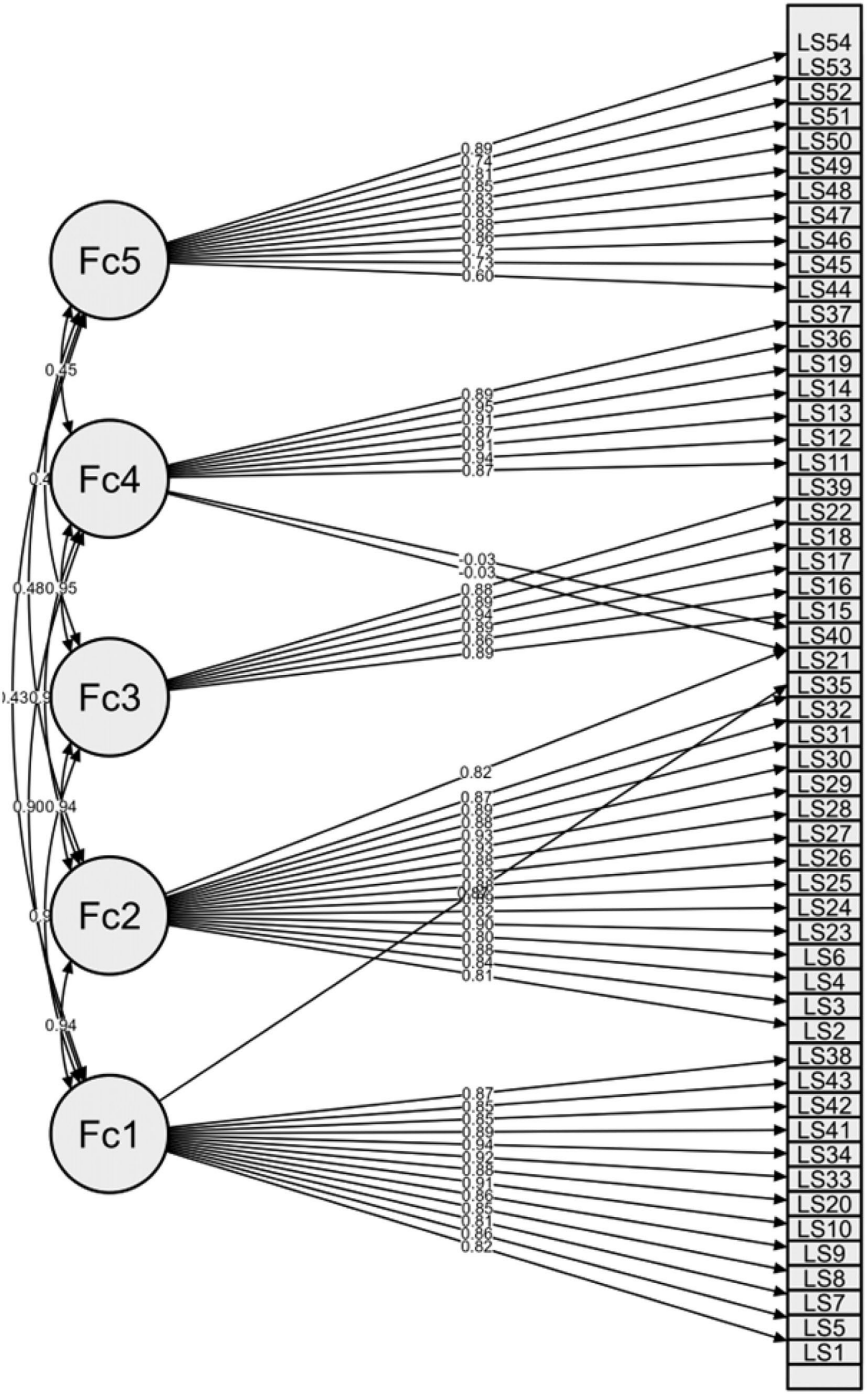
Model plot for Transformational Leadership Scale.

The analysis showed that the 5‐factor TLS model was well fitted to the data, *χ* ^2^ (1313) = 2990.57; *p* < 0.001; CFI = 0.998; RMSEA = 0.055 [90% CI: 0.052; 0.057]; SRMR = 0.041. Table 2 and Figure 1 presents basic descriptive statistics, item‐scale correlations and factor loading values; Table 3 shows CFA results.

**TABLE 2:** Descriptive statistics, item‐scale correlations and factor loading values for the 5‐factor Transformational Leadership Scale structure.

No.	Items	Scale	*M*	SD	Item‐Scale correlations	Λ
LS1	Listens to the opinions of the employees when making decisions	Leadership ethics	3.26	1.24	0.78	0.83
LS5	He/She is fair in issues concerning professional decisions regarding each worker		2.94	1.36	0.83	0.87
LS7	Is friendly towards the personnel		3.56	1.24	0.78	0.81
LS8	Respects the workers' rights		3.57	1.24	0.81	0.85
LS9	Is reliable at work		3.71	1.19	0.79	0.87
LS10	Appreciates each worker		3.06	1.36	0.87	0.91
LS20	Is fair in the issues of workload		3.10	1.36	0.79	0.88
LS21	Is fair when planning shift work		3.25	1.33	0.76	0.87
LS33	Is genuinely interested in the well‐being of workers		3.07	1.44	0.84	0.92
LS34	Promotes cooperation in the department by providing a good example		3.10	1.46	0.87	0.94
LS38	Is positive towards workers from various age groups		3.43	1.33	0.81	0.87
LS41	It is easy to appreciate his/her contribution to work		3.29	1.28	0.83	0.90
LS42	His/her contribution to work is appreciated by the whole personnel in the department		3.05	1.27	0.80	0.86
LS43	Is a respected leader also outside of the department		3.14	1.31	0.80	0.86
LS2	Makes decisions basing on currently available knowledge	Nursing process management	3.57	1.24	0.75	0.82
LS3	Explains his/her decisions		3.29	1.30	0.76	0.84
LS4	Makes professional decisions in a logical way		3.31	1.27	0.80	0.88
LS6	He/she provides information about decisions and their consequences quickly		3.32	1.21	0.74	0.81
LS23	Manages in a way which allows efficient work		3.17	1.29	0.84	0.90
LS24	Is goal‐oriented		3.42	1.21	0.79	0.82
LS25	Sets ambitious professional goals		3.08	1.32	0.84	0.89
LS26	Provides high quality care in the department		3.45	1.27	0.84	0.88
LS27	Instructs to pay attention to safety at work		3.62	1.22	0.78	0.83
LS28	Increases work effectiveness with uniform work methods		3.30	1.26	0.85	0.88
LS29	Employs the knowledge obtained during the evaluation of work in the department in work organisation		3.30	1.25	0.88	0.93
LS30	Motivates to improve work in the department basing on knowledge obtained from the evaluation of work in the department		3.24	1.27	0.89	0.93
LS31	Provides instructions concerning the assessment of work results		3.13	1.26	0.83	0.88
LS32	Welcomes the prospect of long‐term activity		3.14	1.26	0.84	0.89
LS35	Selects tasks which are suitable for each worker		3.09	1.32	0.77	0.87
LS40	Shares his/her views and opinions courageously when cooperating with other specialists		3.34	1.30	0.73	0.80
LS15	Provides feedback on the work performed on a regular basis	Feedback and rewards	3.08	1.31	0.81	0.89
LS16	Rewards workers for development		2.64	1.34	0.82	0.86
LS17	Plans work so as to provide the possibility of development to everybody		3.01	1.36	0.85	0.89
LS18	Provides feedback in a manner which motivates to develop further		2.94	1.36	0.89	0.94
LS22	Is fair when rewarding for work		2.94	1.32	0.80	0.90
LS39	Uses personal skills and abilities of workers		3.29	1.30	0.74	0.88
LS11	Informs about the possibilities of professional development	Professional development	3.19	1.39	0.81	0.87
LS12	Motivates each worker to develop		3.08	1.38	0.88	0.94
LS13	Encourages lifelong learning		3.18	1.36	0.85	0.91
LS14	Discusses the possibilities of professional development with each worker regularly during employee assessment		2.68	1.37	0.77	0.87
LS19	Is fair in educational issues		3.16	1.33	0.83	0.91
LS21	Is fair when planning shift work		3.25	1.33	0.68	−0.03
LS36	Encourages everybody to pursue personal development at work		3.09	1.34	0.87	0.95
LS37	Provides opportunity to develop to each worker in the department		3.22	1.34	0.81	0.89
LS40	Shares his/her views and opinions courageously when cooperating with other specialists		3.34	1.30	0.67	−0.01
LS44	Is strong in his/her position	Nursing directors	3.38	1.22	0.58	0.60
LS45	His/her status is equal to that of other members of the managing personnel		2.93	1.16	0.70	0.73
LS46	Shares his/her views and opinions courageously when cooperating with other specialists		2.95	1.15	0.73	0.73
LS47	Is just when making decisions		2.57	1.11	0.73	0.86
LS48	Employs evidence‐based knowledge in decision making		2.87	1.07	0.76	0.88
LS49	Is reliable at work		3.06	1.07	0.76	0.83
LS50	Is characterised by understanding towards nursing personnel of different ages		2.77	1.20	0.73	0.83
LS51	Has precise views on the issue of work quality improvement		2.98	1.10	0.78	0.85
LS52	Motivates and supports the director of the facility in improving work organisation		2.96	1.05	0.75	0.81
LS53	Plays a significant role in making strategic decisions in the hospital		2.97	1.10	0.68	0.74
LS54	Plays a significant leadership role as regards the unification of working conditions in the hospital		2.73	1.16	0.79	0.89

*Note:* The test items LS21 (for the leadership ethics) and LS40 (for the nursing process management) are also included in the professional development scale.

Abbreviations: *M*, mean; SD, standard deviation, Λ, factor loading values.

**TABLE 3:** Confirmatory factor analysis results.

Factor	Indicator	Estimate	SE	*z*‐value	*p*	95% confidence interval	
						Lower	Upper
Leadership ethics	LS1	0.822	0.005	167.927	< 0.001	0.812	0.831
	LS5	0.865	0.005	189.398	< 0.001	0.856	0.874
	LS7	0.811	0.005	158.235	< 0.001	0.801	0.821
	LS8	0.853	0.005	177.714	< 0.001	0.844	0.862
	LS9	0.863	0.005	177.771	< 0.001	0.853	0.872
	LS10	0.909	0.004	215.337	< 0.001	0.901	0.917
	LS20	0.875	0.005	189.642	< 0.001	0.866	0.884
	LS21	0.869	0.066	13.137	< 0.001	0.739	0.998
	LS33	0.920	0.004	227.807	< 0.001	0.912	0.928
	LS34	0.937	0.004	240.060	< 0.001	0.929	0.945
	LS41	0.890	0.004	210.021	< 0.001	0.882	0.898
	LS42	0.854	0.005	182.364	< 0.001	0.844	0.863
	LS43	0.854	0.005	183.322	< 0.001	0.845	0.863
	LS38	0.871	0.005	187.055	< 0.001	0.862	0.880
Nursing process management	LS2	0.813	0.005	159.043	< 0.001	0.803	0.823
	LS3	0.841	0.005	174.566	< 0.001	0.832	0.850
	LS4	0.876	0.005	193.159	< 0.001	0.867	0.885
	LS6	0.804	0.005	161.310	< 0.001	0.794	0.813
	LS23	0.903	0.004	222.317	< 0.001	0.895	0.911
	LS24	0.822	0.005	181.195	< 0.001	0.814	0.831
	LS25	0.890	0.004	217.770	< 0.001	0.882	0.898
	LS26	0.877	0.004	204.958	< 0.001	0.869	0.886
	LS27	0.826	0.005	166.757	< 0.001	0.816	0.835
	LS28	0.877	0.004	212.270	< 0.001	0.869	0.885
	LS29	0.929	0.004	252.751	< 0.001	0.922	0.937
	LS30	0.933	0.004	256.452	< 0.001	0.926	0.940
	LS31	0.883	0.004	218.233	< 0.001	0.875	0.891
	LS32	0.887	0.004	210.337	< 0.001	0.879	0.895
	LS35	0.870	0.004	200.582	< 0.001	0.862	0.879
	LS40	0.820	0.088	9.366	< 0.001	0.649	0.992
Feedback and rewards	LS15	0.886	0.005	182.582	< 0.001	0.876	0.895
	LS16	0.863	0.005	177.960	< 0.001	0.854	0.873
	LS17	0.889	0.005	190.482	< 0.001	0.880	0.898
	LS18	0.940	0.004	220.693	< 0.001	0.932	0.948
	LS22	0.895	0.005	187.818	< 0.001	0.885	0.904
	LS39	0.881	0.005	174.966	< 0.001	0.871	0.891
Professional development	LS11	0.871	0.005	181.014	< 0.001	0.862	0.881
	LS12	0.939	0.004	229.218	< 0.001	0.931	0.947
	LS13	0.907	0.004	214.300	< 0.001	0.899	0.915
	LS14	0.869	0.005	168.548	< 0.001	0.859	0.879
	LS19	0.911	0.005	194.945	< 0.001	0.902	0.920
	LS21	−0.026	0.068	−0.389	0.697	−0.159	0.106
	LS36	0.950	0.004	227.023	< 0.001	0.942	0.958
	LS37	0.893	0.005	190.327	< 0.001	0.884	0.902
	LS40	−0.030	0.090	−0.331	0.740	−0.207	0.147
Nursing directors	LS44	0.598	0.011	56.065	< 0.001	0.577	0.619
	LS45	0.728	0.010	74.165	< 0.001	0.709	0.748
	LS46	0.731	0.010	76.367	< 0.001	0.712	0.750
	LS47	0.858	0.009	95.661	< 0.001	0.840	0.875
	LS48	0.878	0.009	101.185	< 0.001	0.861	0.895
	LS49	0.825	0.009	94.079	< 0.001	0.808	0.843
	LS50	0.833	0.009	93.410	< 0.001	0.815	0.850
	LS51	0.853	0.009	97.414	< 0.001	0.836	0.871
	LS52	0.813	0.009	90.257	< 0.001	0.796	0.831
	LS53	0.737	0.009	79.001	< 0.001	0.719	0.755
	LS54	0.890	0.009	102.065	< 0.001	0.873	0.907

*Note:* The test items LS21 (for the leadership ethics) and LS40 (for the nursing process management) are also included in the professional development scale.

### 4.3 Reliability and Convergent Validity

The analysis confirmed the reliability and convergent validity (AVE > 0.5) of the scales (Table 4).

**TABLE 4:** Transformational Leadership Scale reliability.

	The number of test items	McDonald's *ω*	Cronbach's *α*	CR	AVE
Leadership ethics	14	0.968	0.968	0.977	0.766
Nursing process management	16	0.971	0.971	0.980	0.751
Feedback and rewards	6	0.940	0.939	0.960	0.799
Professional development	9	0.949	0.948	0.924	0.639
Nursing directors	11	0.935	0.934	0.951	0.640

*Note:* McDonald's *ω* (> 0.7); Cronbach's *α* (> 0.7); CR, composite reliability (> 0.6); AVE, average variance extracted (> 0.5).

Table 5 presents correlations between the TLS scales. They are positive and strong, indicating that the higher the score obtained on one scale, the higher the score obtained on the other scales.

**TABLE 5:** Correlation matrix between Transformational Leadership Scale sub‐scales.

	1	2	3	4	5
1. Leadership ethics	—				
2. Nursing process management	0.94***	—			
3. Feedback and rewards	0.93***	0.94***	—		
4. Professional development	0.90***	0.92***	0.95***	—	
5. Nursing directors	0.44***	0.49***	0.48***	0.46***	—

*** *p* < 0.001.

## 5 Discussion

The study used a translation process that allowed the achievement of the highest quality of translation, cultural adaptation and semantic equivalence (Chen and Boore 2010). At this stage, no difficulties were identified and the translated instrument seems clear, understandable and both linguistically and culturally appropriate. To ensure high quality translation, translators acted fully independently to avoid misconceptions and duplication (John et al. 2006). In addition, an expert panel and a monolingual test confirmed the feasibility of the questionnaire, recognising that all items were relevant, understandable and appropriate, substantively correct for use by nurses. Ultimately, no significant differences were identified in the translation; only a few synonyms were noted, which were discussed in the expert group. A consensus was reached and supporting information for the respondent was added to the information section of the survey to facilitate better identification of the persons being assessed in the TLS.

CFA proved that the adopted theoretical TLS model was well fitted to the test data. The chi‐square was statistically significant, which is common in large samples because the test is highly sensitive to sample size. Therefore, model fit was interpreted based on approximate fit indices (CFI, RMSEA, SRMR), which indicated a good fit (Hu and Bentler 1999). This means that the provision of special indices of model adjustment was confirmed assuming the existence of a certain number of factors, on the basis of which it was possible to verify the validity of the adopted theoretical model to the tested data (DeVon et al. 2007). As regards two items in the Professional development scale (LS21 and LS40), the values of the factor loadings were negative. Both items were part of other scales, indicating that they were more strongly associated with those scales. Item‐scale correlations indicated that these items were related to both the Professional development and other scales, that is, the correlations were above 0.6 for item LS21—Leadership Ethics and for item LS40—Nursing process management. Excluding or adding these items did not affect the reliability of the scale (it did not increase or decrease it), so the original structure of the tool was retained. In future studies using this tool, researchers should consider applying reverse coding to these items, which may increase the validity and reliability of the scale, as well as the variability and diversity of the data. These items do not affect the measured TL construct, so removing items LS21 and LS40 from the subscales in which they achieved negative item‐scale correlations will avoid scale redundancy. It allowed the adoption of a 5‐factor model with 54 items, as in the primary study (Eneh et al. 2012). This means that the structure adopted by the Finnish researchers was also well adjusted to the structural model in the present study, which confirms the high quality of the TLS test tool. The adopted factor model and the items included reflect the full content of the measured construct. Therefore, the adopted structure of the questionnaire, using isolated factors and their variables, may be used as a reliable research tool for identifying and measuring TLs in a similar study group (Gray and Grove 2021).

Internal consistency for the TLS was confirmed. The use of three indices to assess reliability was aimed at eliminating possible errors that could cast doubt on the credibility of the tool. Studies indicated that Cronbach's *α* has limitations, for example, ones that are related to the overestimation of Cronbach's *α* in calculations for an entire scale composed of many subscales (Nunnally and Bernstein 1994). The specificity of this coefficient in terms of the test sample means that it should be calculated for each test sample separately (Waltz et al. 2005). Depending on the purpose of the study, the acceptable values of the index may vary from > 0.60, > 0.70, or > 0.90 (Sousa and Rojjanasrirat 2011). Due to the limitations of Cronbach's *α*, McDonald's *ω* and CR coefficients were calculated to assess the reliability of the tool. In the case of multivariate tests, they allow the assessment of both the reliability of the entire test and the impact of the general factor and subfactors on the reliability of the test (Ciżkowicz 2018). In addition, a clearly defined relationship between the *ω* coefficients and the measurement model reduces the likelihood of their misinterpretation (Taber 2018). Both can be used to assess the reliability of the instrument, and high‐quality results may be obtained. In the original and subsequent studies using TLS, reliability was also confirmed using Cronbach's *α* (Eneh et al. 2012; Kvist et al. 2013). This demonstrates the high reliability of the tool and the high internal consistency of the TLS, making it a reliable tool for assessing the TL in similar groups.

The analysis confirmed convergent validity, which means that the indices were consistent with other measures of the same construct and described the studied construct in a reliable way (Souza et al. 2017). In addition, strong and positive correlations between the sub‐scales indicated that they were statistically significant and that the constructs were interrelated. Those correlations indicated a consensus in the measurement of the adopted construct by the determined variables, and a change in one was systematically associated with changes in the others. It confirmed a strong correlation between the variables in the structure of the TLS questionnaire and the TL study, thereby confirming its reliability (Beck and Polit 2021).

Correlation values for ‘Leadership Ethics’, ‘Nursing process management’, ‘Feedback and rewards’ and ‘Professional development’ factors were between 0.90 and 0.95, indicating a high correlation with the TL construct. The ‘Nursing director’ factor, which presented lower correlation values (0.44 to 0.48) suggesting moderate correlation with TL. This factor was related to the nursing director's assessment of management, while the first four factors relate to the nurses' direct supervisor (coordinating nurse/charge nurse). In the context of Polish nursing and work organisation, it is the direct supervisor who has the most influence on creating and displaying leadership style among nursing staff which may explain the weaker association of the ‘Nursing director’ factor with the rest of the scale.

In the literature, several instruments are used to assess transformational leadership, including the Multifactor Leadership Questionnaire (MLQ) and the Transformational Leadership Questionnaire (TLQ). Although the MLQ is widely regarded as the gold standard due to its strong psychometric properties, it does not focus exclusively on transformational leadership and is less tailored to the clinical nursing context (Bass and Avolio 1996). The TLQ, while specifically designed to measure transformational leadership, demonstrates moderate reliability and is characterised by variability across different settings, with better applicability in public sector organisations than in clinical nursing environments (Alban‐Metcalfe and Alimo‐Metcalfe 2000). In comparison, the Transformational Leadership Scale (TLS) focuses solely on transformational leadership and appears more closely aligned with the realities of nursing practice. However, it should be noted that further cross‐cultural adaptation and validation are needed to confirm its applicability across diverse settings.

The present study contributes to the existing international literature by providing a psychometrically sound instrument for assessing TL among ICU nurses in Poland. This may facilitate further research using the TLS in national studies and support future cross‐cultural comparisons. Additionally, the findings may inform the development of strategies for the implementation of TL in Poland and potentially in broader international contexts.

### 5.1 Limitations of the Work

The use of convenience sampling is a limitation of this study, as it may lead to selection bias, which may limit the generalisability of the findings. However, the high participation rate and the use of clear inclusion criteria allowed for the correct identification and characterisation of the sample and may support the reproducibility of the results (Gray and Grove 2021). The study did not include calculations related to the sample size, as it was determined that the sample size would depend on the number of people per item on the scale (5–10 people/item), that is, 8 respondents per item in the present study (Boateng et al. 2018). A high proportion of sociodemographic data were missing. Although handled using full information maximum likelihood (FIML) under the assumption of missing at random (MAR), this may introduce bias and limit the generalisability of participant characteristics. Another limitation may be associated with the inability to calculate the response rate due to the use of an online questionnaire. Stress that could occur in the study participants was another difficulty which was potentially related to the assessment by the immediate supervisor and the fear of possible consequences.

### 5.2 Recommendations for Further Research and Relevance to Clinical Practice

The TLS is a reliable and psychometrically valid tool for examining TL and evaluating interventions to improve TL among intensive care nurses in Poland. Implementing the TLS into daily practice could be a starting point for implementing and developing new management approaches in ICU, highly recommended in health care to improve job efficiency, job satisfaction and quality of care. Further research should include validating the scale in settings other than intensive care nursing and testing the psychometric properties of the tool in other countries and cultures.

## 6 Conclusion

The findings suggest that the TLS is a linguistically and culturally appropriate tool for Polish settings and may be used to assess TL levels among ICU nurses. It demonstrates satisfactory psychometric properties, indicating its potential usefulness in research involving Polish nurses.

## Author Contributions

**Katarzyna Lis:** conceptualisation (lead); methodology (lead), investigation (lead), formal analysis (lead); writing – original draft (lead); writing – review and editing (equal); supervision (lead). **Natalia Sak‐Dankosky:** conceptualisation (equal); methodology (equal), formal analysis (equal); writing – original draft (supporting); writing – review and editing (supporting), supervision (supporting). **Bożena Czarkowska‐Pączek:** conceptualisation (equal); methodology (supporting), formal analysis (supporting); writing – original draft (supporting); writing – review and editing (supporting); supervision (supporting). All authors read and approved the final manuscript.

## Funding

The authors have nothing to report.

## Disclosure

Statistics: The authors have checked to make sure that our submission conforms as applicable to the Journal's statistical guidelines described here. The statistics were checked prior to submission by an expert statistician: Marta Formela, email: mformela@pogotowiestatystyczne.pl. The author(s) affirm that the methods used in the data analyses are suitably applied to their data within their study design and context, and the statistical findings have been implemented and interpreted correctly. The author(s) agrees to take responsibility for ensuring that the choice of statistical approach is appropriate and is conducted and interpreted correctly as a condition to submit to the Journal.

## Conflicts of Interest

The authors declare no conflicts of interest.

## Data Availability

The data supporting this study's findings are available from the corresponding author upon request.
